# Hands Deserve Better: A Systematic Review of Surgical Glove Indicator Systems and Identification of Glove Perforation

**DOI:** 10.3390/jcm14227977

**Published:** 2025-11-11

**Authors:** Kurt R. Wharton, Robert G. Sawyer, Andreas Enz, Jessica Bah-Rösman, C. Tod Brindle

**Affiliations:** 1 Department of Obstetrics and Gynecology, Oakland University William Beaumont School of Medicine, Rochester, MI 48309, USA; kurt.wharton@corewellhealth.org; 2 Homer Stryker MD School of Medicine, Western Michigan University, Kalamazoo, MI 49007, USA; robert.sawyer@wmed.edu; 3 Department of Orthopedics, Helios Kliniken Schwerin and Medical School Hamburg, 19055 Schwerin, Germany; 4 Department of Orthopedics, University Medicine Rostock, 10857 Rostock, Germany; 5 Global Medical Affairs, Mölnlycke Health Care, 43121 Mölndal, Sweden; jessica.bahrosman@molnlycke.com; 6 Independent Researcher, Norcross, GA 30092, USA; c.todbrindle@gmail.com

**Keywords:** indicator system, surgical site infection, gloving practice, surgical gloves, glove breach

## Abstract

**Background:** The maintenance of an aseptic barrier between the surgical team and patient aids in the prevents the exposure of the patient to pathogens. Variations in gloving practice may have safety implications due to glove failure. Indicator gloving, where two pairs of different colored gloves are worn, is thought to add protection as it alerts the wearer to a breach. The aim of this systematic review and meta-synthesis was to assess the evidence in the literature on the benefit of indicator systems versus other forms of double gloving on puncture identification during surgery. **Methods:** A four-arm, parallel, systematic review of the literature was undertaken in accordance with the PRISMA statement using four distinct research questions regarding glove fit, double gloving, indicator gloves, and the association between glove damage and glove change frequency. Searches on PubMed, Embase, Cochrane Collaboration of Systematic Reviews and Metanalyses, and Google Scholar were performed between 1 May 2022 and 24 January 2023. Studies were assessed for eligibility against pre-defined inclusion and exclusion criteria. Risk of bias was determined using multiple assessment tools. **Results:** This systematic review included 32 studies, 10 of which were high-quality Level IA trials, alongside multiple observational analyses. Across studies, indicator glove systems consistently demonstrated a two- to six-fold higher detection rate of glove perforations compared with standard double-gloving using identical glove colors. The majority of studies confirmed that early perforation identification significantly reduces intraoperative contamination risk and improves maintenance of the aseptic barrier. However, evidence regarding the direct impact on surgical site infections remains limited due to study heterogeneity. **Conclusions:** The use of an indicator glove provides a two- to six-fold higher rate of glove perforation detection, compared to two standard gloves of the same color, therefore aiding the maintenance of the aseptic barrier between surgical team members and patients. Further research into the impact of double gloving on the incidence of surgical site infections is required.

## 1. Introduction

Surgical gloving practice has progressed past a series of milestones over the last 150 years. Those milestones were the result of specific technological advances in response to and guided by the observation and experience of surgeons and nurses. Hernigou (2022) reported on this bizarre journey from lack of asepsis or gloves in the time of Lister and Pasteur, through protection against carbolic acid for cleaning instruments, to general acceptance and use by the 1950s [1]. Interestingly, in studying historical photographs of active operating theaters, he identified that only 30% of surgeons wore gloves by 1900, while specialties like trauma surgery did not experience rates of over 30% use until around 1940. World War II brought about a near 75% adoption of surgical glove use in addition to the first reports of double gloving [1,2]

The Ansell Corporation introduced the first disposable gloves in 1964. With the adoption of latex, Maibach and Johnson in 1975 [3] first reported on the risk of allergic contact dermatitis with exposure to latex proteins, which led to the search for raw material changes. An increased interest in the topic was explored following the increase in personal protective equipment use in the 1980s after the start of the human immunodeficiency virus (HIV) pandemic. While latex remains the preferred material for surgical gloves in much of the world, the 1990s saw the introduction of polyisoprene. This polymer naturally occurs in plants, can be created synthetically, and was introduced to remove the risk of latex-specific protein allergy in surgical gloves by creating a synthetic form of latex. Various forms of polyisoprene exist in the surgical gloves of today, with manufacturers continuing to innovate to reduce chemical accelerators such as thiazoles, thiurams, carbamates, thioureas, and diphenylguanidine which may similarly lead to contact dermatitis. In the early 2000s, a new material was introduced into surgical gloves, polychloroprene. This synthetic elastomer provided an alternative to natural rubber latex while removing additional chemical accelerators and preservatives for the most sensitive clinicians.

**Figure 1:**
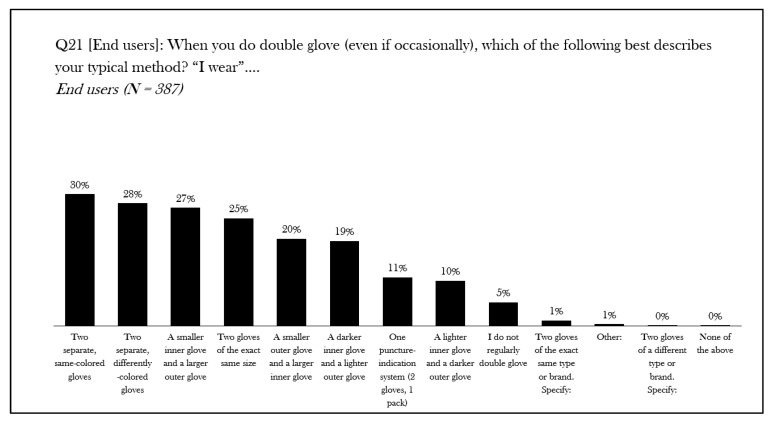
Ethnographic research study results on double gloving methods: all users. An ethnographic qualitative research study of 512 surgeons, nurses, and scrub technicians from Germany, Italy, India, and the United States examined the practice, perception, and experience of operating personnel and gloving practice. The chart shows the results for the research question ‘when you do double glove (even if occasionally), which of the following best describes your typical method? “I wear…”’. Results are expressed as percentage response [14]. N = number, Q = question.

**Figure 2:**
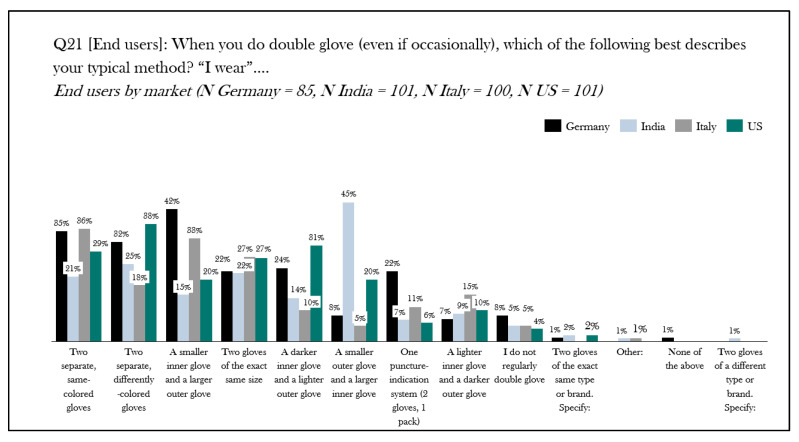
Ethnographic research study results on double gloving methods: all users by country. An ethnographic qualitative research study of 512 surgeons, nurses, and scrub technicians from Germany, Italy, India, and the United States examined the practice, perception, and experience of operating personnel and gloving practice. The chart shows the results for the research question ‘‘when you do double glove (even if occasionally), which of the following best describes your typical method? “I wear…”’. Results are expressed as percentage response and by country [14]. N = number, Q = question, US = United States.

Over the last thirty years, there have been increasing reports on the perforation rate of surgical gloves for operating room personnel [4,5,6,7,8,9,10,11,12]. Moreover, the rate that these breaches go unnoticed has been reported to be as high as 90% [13]. In 1993, Mölnlycke Health Care was the first to introduce puncture indicator^®^ technology, a double-gloving system composed of specially designed, colored undergloves with a white over-glove, specifically designed to illuminate when the aseptic barrier of the outer glove had been breached. Since then, other manufacturers have introduced their own indicator systems to the market. Surprisingly, despite its introduction over 30 years ago, the adoption of this technology appears to be isolated to specific geographic regions. In an ethnographic research study of 512 surgeons, nurses, and surgical technicians across the US, India, Germany, and Italy, the practice of double gloving remains relatively low [14]. Moreover, double-gloving specific practice, e.g., wearing two of the same gloves, a specialized indicator system, or some other combination of gloves, is highly variable (Figure 1). This practice appears to differ by country (Figure 2).

Stearns and colleagues recently reported a systematic review of the literature comparing single-gloving to double-gloving practice on the risk of complications for patients and providers [15]. Double gloving was found to be superior to single gloving in maintaining an aseptic barrier; the question for clinicians is if there is existing evidence to determine how double gloving should be performed for maximum benefit. Specifically for this article, what is the benefit of an indicator system versus other forms of double gloving on puncture identification during surgery?

In order to examine the relationship of gloving practice on the ability to identify perforations during surgery, a systematic review of the literature was performed. This systematic review was part-three of a four-arm, parallel, systematic review of the literature and Delphi-consensus recommendation project, the results of which will be published separately [16].

The objective of this project was to determine the best available evidence describing four key fundamental principles of surgical gloving practice: glove fit, double gloving, puncture indication, and glove change frequency. The purpose of the research and consensus process is to inform existing and future operating room staff on the importance of appropriate gloving practice to optimize health care provider (HCP) performance and ensure provider and patient safety.

This article specifically reports on part three of this program of research (glove indicator systems) to expand upon existing research limited to provider needle stick injuries [17].

## 2. Materials and Methods

The study was conducted in accordance with preferred reporting items for systematic reviews and meta-analysis protocols (PRISMA) statement [18] and follows the published checklist for reporting. It was registered in the International Platform of Registered Systematic Review and Meta-Analysis Protocols (INPLASY; registration number INPLASY2025100008).

### 2.1. Eligibility Criteria

This systematic review was performed between 1 May 2022 and 24 January 2023. The objective of this study was to determine the best available evidence describing the difference between the use of specialized indicator systems versus the common practice of wearing two standard gloves while double gloving during surgical cases in the operating theater. The purpose of the study was to help inform current surgical praxis and future innovation to protect providers and patients from the risks associated with aseptic barrier breach.

Therefore, the research question for this systematic review is as follows: for health care providers scrubbed into surgical procedures, what is the difference between specialized double-gloving indicator systems compared to wearing two standard gloves on the identification of glove perforation?

To meet the objectives and answer the research question, a pluralistic approach was used to identify all available evidence and determine the highest quality evidence. The year 1980 was used as the cut-off point for including historical studies as latex surgical gloves were first introduced to the market in 1964, with significant advances in present-day raw materials introduced in the late 1990s (polyisoprene) and into the 2000s (polychloroprene). However, 1980 was used to avoid historical bias associated with the HIV/AIDS pandemic which brought considerable attention to personal protective equipment, the performance of surgical gloves as an essential barrier in the operating room, and reports of double gloving to avoid a complication (HIV transmission). While advances in the field are on-going for manufacturers of surgical gloves, articles were considered if they were published between 1 January 1980 through 1 January 2023. Additional inclusion criteria included studies of surgeries performed in adult and pediatric populations, any surgical specialty, and acute-care operating rooms. Studies were eligible if they investigated surgical glove use with indicator systems, double gloving, or glove perforation identification in clinical or experimental surgical settings. Because the first commercial double-glove indicator system was introduced in 1993, all included studies were published from that year onward. No language restrictions were applied. All articles were assigned to reviewers with native language capabilities or were translated to English using a third-party translation software. The hierarchy of the study types included were as follows:

- Systematic reviews and meta-analysis;
- Randomized controlled clinical studies;
- Quasi-experimental studies;
- Cohort with control designs;
- Case-controlled designs;
- Observational studies;
- Healthy volunteer studies;
- In vitro studies.

Studies were excluded if they reported testing of non-sterile medical exam gloves, and/or they involved any of the following:

- Dentistry or orthodontics;
- Local, interventional procedures performed outside of the operating theater (e.g., emergency department, etc.);
- In vitro studies or simulated surgeries;
- Veterinary surgery;
- From 1979 or older;
- No abstract available;
- “Antimicrobial glove” studies;
- Narrative reviews, commentaries, and letters to the editor;
- Quality improvement projects, case studies, or case series.

### 2.2. Information Sources

Four databases were used to identify prospective articles, including PubMed, Embase, Cochrane Collaboration of Systematic Reviews and Metanalyses, and Google Scholar. Secondary searches of all included study references were completed by the reviewers manually. All non-open access articles were purchased through RightFind™ (Copyright Clearance Center, Danvers, MA, USA).

### 2.3. Search Strategy

The literature search was designed according to the PICO (Population, Intervention, Comparison, Outcome) framework. The Population included surgical team members using sterile gloves; the Intervention was the use of double gloving with an indicator system; the Comparison was standard double-gloving with identical glove colors; and the Outcome was the detection of glove perforation or maintenance of the aseptic barrier.

**Figure 3:**
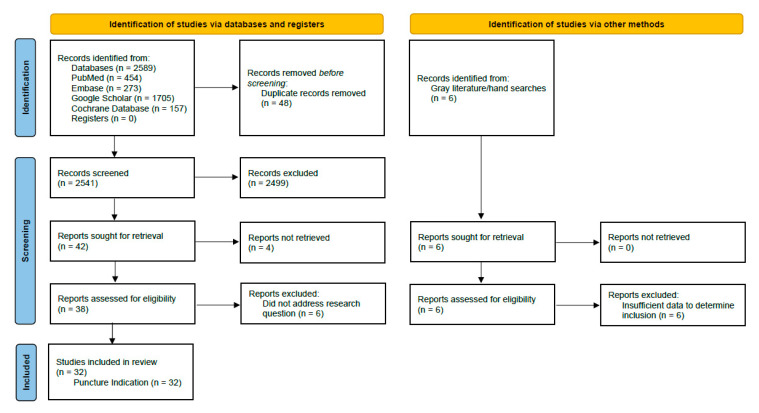
PRISMA flow chart. PRISMA, preferred reporting items for systematic reviews and meta-analysis.

The search strategy was defined through a combination of four domains and initial key terms, with a second selection of terms added following initial search results (Table S1). Domain one defined terms for the population and setting, domain two identified terms associated with the device (glove), domain three explored relevant terms for glove integrity, and domain four covered terms associated with indicator systems. Four weeks of initial keyword searches were performed to help refine the final study keywords to improve precision of abstract identification. The primary search limits for the databases included a custom date range of 1980–2023. Search limitations including Boolean phrase NOT terms were selected based on initial keyword search findings. A representative PubMed search string was (“surgical gloves” OR “double gloving” OR “indicator gloves”) AND (“perforation detection” OR “barrier integrity” OR “contamination risk”). All search strategies are provided in Supplementary Table S1. Results of the search strategy are reported using the PRISMA study flow chart (Figure 3).

### 2.4. Data Management and Selection Process

Two reviewers (KW and RS) independently provided screening of all identified abstracts for eligibility. Critical appraisal was carried out by monthly meetings to verbally discuss inclusion versus exclusion designation. When there were disagreements with any aspect of the process regarding identified studies, the reviewers presented their rationale for selection and determined if an agreement could be made on inclusion. If agreements could not be made, a third researcher (AE) would be called in to render a final decision. The reviewers used an encrypted sharing platform via Microsoft Teams which was only accessible by invitation from the reviewers. Following agreement on which abstracts to send for full review, the reviewers independently read all full-text manuscripts and subsequently met to determine the final studies for analysis. Meta-analysis was not a goal of this systematic review due to methodological heterogeneity among the studies across a broad range of potential complications. Meta-synthesis of study outcomes was utilized. The data collection process for the extraction of each study was performed by the researchers in tandem over multiple virtual and in-person meetings where data reporting was categorized by outcome domains and transferred along with study demographics into evidence tables.

### 2.5. Data Items

The population of “health care provider” includes all operating room personnel, specifically, the following: surgeon, resident or assistant surgeon, surgical technician, scrub nurse, first assist, and physician assistant. The device was defined as a surgical glove manufactured of natural rubber latex, polyisoprene, polychloroprene, or a combination thereof. Double gloving was defined as wearing an additional pair of gloves during the operative case. While double gloving is generally considered to refer to the wearing of two pairs of gloves, studies reporting the use of three gloves were also captured. The topics of allergic or contact dermatitis associated with provider reaction to various raw materials or associated accelerators were not within the scope of this review as this has previously been addressed in the literature ad nauseam.

### 2.6. Risk of Bias

Risk of bias was determined utilizing the Ottawa–Newcastle Quality Assessment Form for non-randomized observational and cohort studies [19], the revised Cochrane risk of bias tool for randomized studies (ROB 2.0) [20], and the Robis risk of bias tool in systematic reviews (ROBIS 1.2) [21].

### 2.7. Data Synthesis

Due to methodological and outcome heterogeneity across the included studies, a quantitative meta-analysis was not feasible. However, heterogeneity among comparable subgroups was descriptively assessed and where overlapping data were available, an exploratory I^2^ statistic was calculated to estimate between-study variability. These analyses consistently indicated high heterogeneity (I^2^ > 75%), confirming that pooling of results would not yield meaningful summary estimates. Therefore, data synthesis will focus on reporting findings from studies comparing the risk of provider and patient complications between two types of gloving procedures on the rate of aseptic barrier breach, health care provider exposure or injury, and reports of surgical site infection or wound complications.

### 2.8. Meta-Bias

Critical appraisal of studies was performed via direct discussion between the reviewers at monthly meetings to examine appropriateness for inclusion, study design interpretation, and inherent bias.

### 2.9. Confidence in Cumulative Evidence

Overall confidence in the quality of study evidence and recommendations for clinical practice was graded using the quality of evidence determination with the *Johns Hopkins Nursing Evidence-Based Practice* (Appendix C) evidence level and quality guide [22].

## 3. Results

Figure 3 represents the scope of the literature search. PubMed, Embase, Cochrane Collaboration of Systematic Reviews and Metanalyses, and Google Scholar identified 2589 records. After the elimination of duplicate articles, the remaining 2541 records were screened by abstract according to the inclusion/exclusion criteria. A total of 2503 studies were excluded from further analysis and 38 records were assessed as suitable for full text review. A total of 32 of these studies were assessed as eligible for inclusion in the review (Table 1 and Table 2). Level of evidence, quality assessments, and risk of bias scores are detailed in Table 3.

### 3.1. Summary of Evidence Prior to Current Review

A Cochrane review by Tanner and Parkinson in 2006 [31] aimed to assess the effect of double gloving on the incidence of surgical site infections. Indicator gloves were assessed as one of the double-gloving systems. Their review identified three studies that addressed indicator glove systems. They concluded that the use of indicator gloves increased the rate of detection of glove perforation in the outer gloves but did not increase the rate of detection of inner glove perforations.

### 3.2. The Effect of Using Indicator Glove Systems on the Rate of Detection of Glove Perforations

Ten level IA studies were identified for inclusion in this review (Table 1). A comprehensive Cochrane review by Tanner and Parkinson (2006) identified three studies that assessed the use of indicator glove systems [31]. After pooling the results of these studies, the authors found that when using indicator gloves, 74% of perforations in the outer glove were detected, compared to 25% in the non-indicator double glove group. There was no difference in the overall frequency of glove perforations nor in the detection of perforations to the inner glove [31].

A level IA systematic review by Tanner and Parkinson (2007) assessed 30 randomized controlled trials that measured glove perforations [32]. Five trials assessed the use of indicator glove systems and were included in a meta-analysis. When an indicator system was used, 77% of glove perforations were detected compared to a detection rate of 21% when standard double-gloving was used. The authors concluded that the use of an indicator system statistically significantly increased the rate of perforation detection [32].

In a level IA randomized controlled trial, Naver and Gottrup (2000) observed that 42% of glove perforations were detected when single gloves were used [29]. When an indicator glove system was used, 78% of glove perforations were detected [29].

A level IA prospective randomized study by Nicolai et al. (1997) identified a significantly higher recognition of glove perforation in the indicator glove group compared to the control group [30]. In the indicator group, 28.3% of perforations were detected compared to a 10.2% detection rate in the control group. The authors recommended the use of indicator gloves in hip and knee arthroplasty [30]. A further level IA prospective randomized study by Avery et al. (1999) identified an overall rate of unnoticed outer glove perforations of 56% [24]. In the double glove group, the rate of glove perforations that remained unnoticed was 81% compared to a rate of 21% in the indicator system gloves. The authors concluded that indicator gloves are particularly useful for surgeries where the operating field is wet [24].

In a level IA observational study, Laine and Aarnio (2001) observed that the level of glove perforations that were noticed when using single gloves was 36.8%, while when using the double gloving indicator system, 86.5% of glove perforations were noticed [27]. A second level IA observational study by Laine et al. (2004) found that when using double indicator gloves, only 10% of glove perforations were not noticed [26]. However, when single gloves were used, 69% of glove perforations were not noticed [26]. A further level IA observational study by Lee (2022) found that 68% of glove perforations were detected when colored indicator undergloves were used, compared to a detection rate of 29% when indicator gloves were not used [28].

Additional studies with a lower strength of evidence and higher bias were also identified, including eight Level II studies, which support the evidence detailed above (Table 2).

## 4. Discussion

This systematic review has identified multiple studies, including a Cochrane review and a systematic review, which detail the benefits of the use of indicator gloves in the detection of glove perforations. The included studies assess the glove perforation rates of multiple different color gloves from multiple manufacturers. Of the 32 articles identified, ten were Level I studies and eight were Level II studies in favor of the use of indicator glove systems due to the increased rates of detection of glove perforation observed. The use of an indicator glove provides between a two- and six-fold higher rate of detection of glove perforation, compared to the use of two standard gloves of the same color [5,24,26,28,29,30,31]. The use of double gloves and an indicator system aid in the maintenance of the aseptic barrier between surgical team members and patients. Further studies are required to assess whether the maintenance of the aseptic barrier by additional glove protection has any impact on the number of surgical site infections observed. Although surgical team members may consider that double gloves have a detrimental impact on manual surgical dexterity, there is currently little evidence in the literature to support this view [31]. As multiple glove indicator systems are available from many suppliers, a glove system fitting session is recommended for surgical team members to determine the available technologies and nuances between manufacturer offerings and ensure optimal glove comfort and surgical performance.

Limitations of this review include the observational, single-center nature of some of the included studies, resulting in generalized findings. There is a need for future research to determine the rate of underglove puncture frequency when an outerglove perforation occurs. There is currently insufficient evidence to determine whether underglove changes are necessary and at what time during surgery a change should be implemented. Further research by manufacturers should consider the development of technologies that allow for the identification of underglove perforations for these systems.

## 5. Conclusions

Indicator glove systems are recommended, as they improve perforation detection two- to six-fold compared to standard double-gloving and help maintain the aseptic barrier without a clear loss of dexterity. Further research should assess their effect on infection rates, optimal change timing, and underglove perforation detection.

## Figures and Tables

**Table 1:** Level IA studies.

Author, Year	Title	Aim	Key Findings
Abdulkarim A. et al., 2015 [23]	The effect of orthopedic surgery on the intrinsic properties of surgical gloves	To study the effect of cement on gloves.	Exposure to cement during orthopedic surgery caused loss of tensile strength of latex and neoprene gloves.
Avery, C. M. E. et al., 1999 [24]	Double gloving and a system for identifying glove perforations in maxillofacial trauma surgery	To determine if a perforation identification system offers additional protection when double gloving.	Standard DG vs. use of indicator glove; Unobserved outer glove perforation rate of 56%; Unobserved inner glove perforation rate of 91.5%; Indicator system had an 81% detection rate; Standard DG had 21% detection rate.
Cox, M. J. et al., 1994 [25]	New advances in electronic devices for hole detection	Describe the design, operation, and performance of electronic glove hole detection devices.	The Surgical Alert Monitor device, in conjunction with gloves that resist hydration, appeared to be a reliable hole detection monitor.
Laine, T. et al., 2004 [26]	Glove perforations in open and laparoscopic abdominal surgery: the feasibility of double gloving	RCT to compare glove perforation rates between single gloving and double gloving with an indicator system (Biogel).	Intra-operative identified perforations: open surgery single glove 31.4%; open surgery double glove 90.3%; laparoscopy single glove 33.3%; laparoscopy double glove 100%; Intra-operative detection is more likely with indicator double gloves, *p* < 0.01.
Laine, T. and Aarnio, P., 2001 [27]	How often does glove perforation occur in surgery? Comparison between single gloves and a double-gloving system	RCT to compare glove perforation rates between single gloving and double gloving with indicator system (Biogel).	In single gloves, 28 perforations were observed from 76 perforations during the surgery (36.84%); in the double-glove system, 77 perforations were observed out of 89 (86.52%); and in the combination group, 9 out of 27 were observed (33.33%; *p* < 0.001); If the duration of the operation was longer than 2 h, 138 perforations out of 1180 gloves (11.69%) were observed; When the duration was less than 2 h, 54 perforations out of 1282 gloves (4.21%; *p* < 0.001) were observed; The risk of glove perforation increases 1.115 (95% confidence interval 1.085 to 1.1469) times for every 10 min of operating time; The perforations were significantly more common in emergency surgeries.
Lee, S. Y., 2022 [28]	What role does a colored under glove have in detecting glove perforation in foot and ankle procedures?	RCT to compare rates of perforation by WLT using two regular gloves or indicator systems.	A 19% glove perforation rate; A 4% rate of under glove perforation; Intra-operative discovery of perforation: 29% with regular gloves, 68% with indicator gloves (*p* < 0.001).
Naver, L. P. and Gottrup, F., 2000 [29]	Incidence of glove perforations in gastrointestinal surgery and the protective effect of double gloves: a prospective, randomized controlled study	RCT to compare Biogel vs. Biogel indicator DG for glove damage	A total of 20% of single and outer gloves were perforated; Blood was found on hands in 13% of cases with single gloves and 2% of double gloves; Intra-operative detection: single gloves 42%, double gloves with indicator 78%.
Nicolai, P. et al., 1997 [30]	Increased awareness of glove perforation in major joint replacement. A prospective, randomized study of Regent Biogel Reveal gloves	RCT to compare rates of glove perforation between standard DG and regent Biogel reveal.	Perforation rates: 19.6% standard glove; 11.0% of Reveal gloves (*p* < 0.01); Intra-operative recognition of perforation: 16.7% standard gloves, 56.5% Reveal gloves (*p* < 0.017).
Tanner, J. and Parkinson, H., 2006 [31]	Double gloving to reduce surgical cross-infection	Determine if double gloving rather than single gloving reduces the number of post-operative or blood borne infections in surgical patients or blood borne infections in the surgical team. Determine if double gloving reduces the number of perforations to the innermost pair of surgical gloves.	Change rates of perforation but detected more perforations intraoperatively of outer gloves; Of perforation but detected more perforations intraoperatively of outer gloves.
Tanner, J. and Parkinson, H., 2007 [32]	Surgical glove practice: the evidence	Systematic review of 30 RCTs; Meta-analysis of 5 trials and 582 gloves.	A total of 52/244 (21%) of standard double gloves detected perforations, vs. 260/338 or 77% with indicator systems; Results were statistically significant.

DG = double gloving; RCT = randomized controlled trial; WLT = water leak method.

**Table 2:** Other included studies.

Author, Year	Title	Key Findings
Brown, J. N., 1996 [33]	Surgeon protection: early recognition of glove perforation using a green under glove	A total of 40 consecutive cases/single surgeon. Green indicator gloves. A total of 26 perforations in 26 outer gloves. A percentage pf 48% of all cases. Outer glove rate 25%. Instruments 59%, exposed bone 30%. Left index finger most common.
de Oliveira, A. C. et al., 2014 [34]	Evaluation of surgical glove integrity during surgery in a Brazilian teaching hospital	Latex single glove study. No indicator. A percentage of 65% of cases; 12 post-op perf rate. No indicator used—suggests that is good to do based on the rate of undetected perforations.
Edlich, R. F. et al., 2003 [35]	Resistance of Double-Glove Hole Puncture Indication Systems to Surgical Needle Puncture	Thicker gloves are more resistant to needlestick puncture. Double gloves are more resistant to needle puncture. Latex single and double glove systems are less resistant to puncture than non-latex and latex single and double gloving.
Edlich, R. F. et al., 2003 [36]	Reliability and Performance of Innovative Surgical Double-Glove Hole Puncture Indication Systems	Indicator systems provide visual evidence of glove puncture.
Edlich, R. F. et al., 2017 [37]	An Update on the Innovative Surgical Double-Glove Hole Puncture Indication Systems: Reliability and Performance	Surgical needle puncture holes were detected by all glove indicator systems assessed.
Edlich, R. F. et al., 2003 [38]	Reducing Accidental Injuries During Surgery	Resistance to needle puncture is significantly greater in double-glove indicator systems than single gloves.
Florman, S. and Burgdorf, M., 2005 [39]	Efficacy of Double Gloving with an Intrinsic Indicator System	Latex—84% of glove perforations were detected, Latex-free—56% of glove perforations were detected.
Grant, C., 2013 [40]	Biogel^®^ Super-Sensitive and Biogel^®^ Indicator glove systems	A combination of Biogel^®^ Super-Sensitive and Biogel^®^ Indicator glove systems was accepted for use by staff following a clinical evaluation.
Hollaus, P. H. et al., 1999 [41]	Glove perforation rate in open lung surgery	Rate of outer glove perforation was 8.9% whilst perforation rate for inner gloves was only 1.13%.
Lee, S. W. et al., 2015 [42]	Perforation of Surgical Gloves during Lower Extremity Fracture Surgery and Hip Joint Replacement Surgery	A total of 25% of gloves were perforated during surgery.
MacIntyre, I. M. C. et al., 2005 [43]	Reducing the Risk of Viral Transmission at Operation by Electronic Monitoring of the Surgeon–Patient Barrier	Glove perforation rate was about 39.8%. The surgeon detected the hole in 11.1%. Only 16.9% of alarms were from glove holes, most were from wet gowns.
Manson, T. T. et al., 1995 [44]	A New Glove Puncture Detection System	More force was required to penetrate the double-glove Biogel Reveal system gloves with a needle than single gloves.
Martinez, A. et al., 2013 [45]	Risk of Glove Perforation with Arthroscopic Knot Tying Using Different Surgical Gloves and High-tensile Strength Sutures	% thin gloves perforated, 6.8% thick gloves. No perforations of both doubled gloves. Suture made no difference.
Sarih, N. M. et al., 2022 [46]	Wearable Natural Rubber Latex Gloves with Curcumin for Torn Glove Detection in Clinical Settings	No change frequency data. Overall glove perforation rate was 11.8%. A total of 21.7% of perforations were noted intra-operatively.
Sayın, S. et al., 2019 [47]	Wearable Natural Rubber Latex Gloves with Curcumin for Torn Glove Detection in Clinical Settings	Successfully added an indicator layer to natural latex gloves. Improved tear strength with coating.
Shek, K. M. Y. and Chau, J. P. C., 2014 [48]	Surgical Glove Perforation Among Nurses in Ophthalmic Surgery: A Case–Control Study	Change frequency undefined. A total of 8% of gloves were perforated, none were detected intra-operatively.
Shimantani M. et al., 2009 [49]	Investigation of the Rate of Glove Perforations in Orthopedic Procedures with an Indicator Underglove System (IUS) in a Japanese Hospital	No comment on glove changes. Outer glove perforation rate 14.1%, inner glove perforation rate 4.5% by WLT. The rate of intra-operative detection was 69.6% for surgeons and 44.4% for nurses.
Sohn, R. L. et al., 2000 [50]	Detection of surgical glove integrity	Electrical conductance testing was more sensitive than water leak test.
Walczak, D. A. et al., 2013 [51]	Evaluation of Surgical Glove Perforation after Laparoscopic and Open Cholecystectomy	No change frequency data given. Overall perforation rate was 8%; perforations more common with laparoscopic surgery.
Wigmore, S. J. and Rainey, J. B., 1994 [52]	Use of Colored Undergloves to Detect Glove Puncture	Outerglove perforation was detected in 29% of outergloves. Indicator gloves had a 97% accuracy of detection rate. The indicator system had a 3% false negative rate.
Wittmann, A. et al., 2009 [53]	Study of Blood Contact in Simulated Surgical Needlestick Injuries with Single or Double Latex Gloving	Punctures clearly identified by green layer indicator glove.
Wittmann, A. et al., 2010 [54]	Comparison of 4 Different Types of Surgical Gloves used for Preventing Blood Contact	Volume of blood transferred by needlestick reduced by a factor of two when using double-glove indicator system.

WLT = water leak test.

**Table 3:** Level of evidence, quality, and risk of bias assessments.

Author, Year	Level of Evidence	Quality	Risk of Bias
Abdulkarim A. et al., 2015 [23]	Level I	Good	Low
Avery, C. M. E. et al., 1999 [24]	Level I	High	Low
Brown, J. N., 1996 [33]	Level II	Good	High
Cox, M. J. et al., 1994 [25]	Level I	Good	Low
de Oliveira, A. C. et al., 2014 [34]	Level II	Good	High
Edlich, R. F. et al., 2003 [35]	Level II	Good	Low
Edlich, R. F. et al., 2003 [36]	Level II	Good	Low
Edlich, R. F. et al., 2017 [37]	Level II	Good	Low
Edlich, R. F. et al., 2003 [38]	Level II	Good	Low
Florman, S. and Burgdorf, M., 2005 [39]	Level II	Good	Low
Grant C., 2013 [40]	Level III	Low	High
Hollaus, P. H. et al., 1999 [41]	Level II	Good	High
Laine, T. et al., 2004 [26]	Level I	High	Some
Laine, T. and Aarnio, P., 2001 [27]	Level I	High	Some
Lee, S. W. et al., 2015 [42]	Level III	Good	High
Lee, S. Y., 2022 [28]	Level I	High	Low
MacIntyre, I. M. C. et al., 2005 [43]	Level III	Good	High
Manson, T. T. et al., 1995 [44]	Level V	Low	High
Martinez, A. et al., 2013 [45]	Level III	Good	High
Naver, L. P. and Gottrup, F., 2000 [29]	Level I	High	Low
Nicolai, P. et al., 1997 [30]	Level I	High	Low
Sarih, N. M. et al., 2022 [46]	Level V	Low	High
Sayın, S. et al., 2019 [47]	Level III	Good	High
Shek, K. M. Yand Chau, J. P. C., 2014 [48]	Level III	Good	High
Shimantani M. et al., 2009 [49]	Level III	Good	High
Sohn, R. L. et al., 2000 [50]	Level V	Low	High
Tanner, J. and Parkinson, H., 2007 [32]	Level I	High	Low
Tanner, J. et al., 2006 [31]	Level I	High	Low
Walczak, D. A. et al., 2013 [51]	Level III	Good	High
Wigmore, S. J. and Rainey, J. B., 1994 [52]	Level III	Good	High
Wittmann, A. et al., 2009 [53]	Level V	Low	High
Wittmann, A. et al., 2010 [54]	Level V	Low	High

## Data Availability

The data that support the findings of this consensus document are available from the corresponding author upon reasonable request.

## Supplementary Materials

The following supporting information can be downloaded at: https://www.mdpi.com/article/10.3390/jcm14227977/s1, Table S1: Domain search terms: indicator systems [55].
